# Bioassay guided purification of the antimicrobial fraction of a Brazilian propolis from Bahia state

**DOI:** 10.1186/1472-6882-9-25

**Published:** 2009-07-30

**Authors:** Myrella Lessio Castro, Walquíria Ribeiro Vilela, Rogéria Cristina Zauli, Masaharu Ikegaki, Vera Lúcia Garcia Rehder, Mary Ann Foglio, Severino Matias de Alencar, Pedro Luiz Rosalen

**Affiliations:** 1 Department of Physiological Sciences, Piracicaba Dental School, University of Campinas (UNICAMP); Avenida Limeira, 901; Piracicaba, SP, 13414-903, Brazil; 2 Department of Agri-Food Industry, Food and Nutrition, Escola Superior de Agricultura "Luiz de Queiroz", University of São Paulo (USP); Avenida Pádua Dias, 11; Piracicaba, SP, 13418900, Brazil; 3 Department of Pharmacy, Federal University of Alfenas, Alfenas, MG, Brazil; Avenida Gabriel Monteiro da Silva, 700; Alfenas, MG, 37130-000, Brazil; 4 Chemical, Biological and Agricultural Pluridisciplinary Research Center (CPQBA), University of Campinas (UNICAMP); C.P. 6171; Campinas, SP, 13081-970, Brazil

## Abstract

**Background:**

Brazilian propolis type 6 (Atlantic forest, Bahia) is distinct from the other types of propolis especially due to absence of flavonoids and presence of other non-polar, long chain compounds, but presenting good *in vitro *and *in vivo *antimicrobial activity. Several authors have suggested that fatty acids found in this propolis might be responsible for its antimicrobial activity; however, so far no evidence concerning this finding has been reported in the literature. The goals of this study were to evaluate the antibacterial activity of the main pure fatty acids in the ethanolic extract and fractions and elucidate the chemical nature of the bioactive compounds isolated from Brazilian propolis type 6.

**Methods:**

Brazilian propolis type 6 ethanolic extract (EEP), hexane fraction (H-Fr), major fatty acids, and isolated sub-fractions were analyzed using high performance liquid chromatography (HPLC), high resolution gas chromatography with flame ionization detection (HRGC-FID), and gas chromatography-mass spectrometry (GC-MS). Three sub-fractions of H-Fr were obtained through preparative HPLC. Antimicrobial activity of EEP, H-Fr, sub-fractions, and fatty acids were tested against *Staphyloccus aureus *ATCC 25923 and *Streptococcus mutans *Ingbritt 1600 using minimum inhibitory concentration (MIC) and minimum bactericidal concentration (MBC).

**Results:**

EEP and H-Fr inhibited the growth of the microorganisms tested; nevertheless, no antimicrobial activity was found for the major fatty acids. The three sub-fractions (1, 2, and 3) were isolated from H-Fr by preparative HPLC and only sub-fraction 1 showed antimicrobial activity.

**Conclusion:**

a) The major fatty acids tested were not responsible for the antimicrobial activity of propolis type 6; b) Sub-fraction 1, belonging to the benzophenone class, was responsible for the antimicrobial activity observed in the present study. The identification of the bioactive compound will improve the development of more efficient uses of this natural product.

## Background

Propolis, a non-toxic resinous hive product collected by *Apis mellifera *bees from various plant sources, has been reported to have several properties that may confer health benefits to humans since ancient times [1,2]. Propolis chemical composition and pharmacological activity might vary significantly depending on its geographic origin and seasonal effect [3-6]. Furthermore, its chemical composition is extremely complex and its flavonoids and (hydroxyl) cinnamic acid derivatives have been widely cited as its biologically active compounds [7-9]. Although these compounds have not been detected in propolis classified as type 6 (from the Atlantic forest, Northeastern Brazil) [10,11], its ethanolic extract and hexane fractions (H-Fr) have shown remarkable antimicrobial activities against pathogens, including mutans streptococci [11-13].

It has been proposed that the antimicrobial activity of type-6 propolis might be due to a high proportion of fatty acids (oleic, palmitic, linoleic, and stearic) identified as part of its chemical composition [13]; nonetheless, such compounds (fatty acids) have not been tested. Therefore, the goals of the present study were to evaluate the antibacterial activity of the main pure fatty acids found in the ethanolic extract and fractions of Brazilian propolis type 6, as well as to elucidate the chemical nature of the bioactive compounds isolated from it.

## Methods

### Propolis samples and fractionation

Crude samples of *Apis mellifera *propolis, originated from the Atlantic forest region, state of Bahia, SL 11°56'31 and WL 38°05'04, in the Northeastern Region of Brazil, classified as propolis type 6 [10] and botanically originated from *Hyptis divaricata *[3], were acquired in May 2006. The ethanolic extract of propolis (EEP) at 20% (w/v) was prepared using aqueous ethanol (80% v/v) according to the literature [13]. The EEP was further fractioned using a liquid-liquid extraction technique with hexane to generate a polar fraction (P-Fr) and H-Fr. The purification grade of the EEP and its fractions was monitored by thin layer chromatography (TLC) using the anisaldehyde reagent (4-methoxy-benzaldehyde, acetic acid, sulfuric acid – 1.0:48.5:0.5), followed by incubation at 100°C for 5 min. Substances were visualized under ultraviolet (UV) light at the wavelengths of 254 nm and 366 nm [6,14]. The EEP, H-Fr, and P-Fr were concentrated in a rotaevaporator at 45°C and yielded 58% (w/w), 14% (w/w), and 11% (w/w), respectively.

### Analytical and preparative HPLC analysis

A Shimadzu Prep 6AD LC system equipped with a SPD-M10Avp photodiode array detector (PDA), an auto injector 10AF, and a fraction collector FRC-10A, was used to perform the HPLC analysis. For the analytical test, diluted solutions of EEP, fractions (H-Fr and P-Fr), and isolated sub-fractions (1, 2, and 3) (1 mg/100 mL) were filtered (Millipore – 0.22 μm), and 10 μL aliquots were injected in a Shimadzu reverse-phase analytical column of 250 mm × 4.6 mm (i.d.) × 5 μm (particle size). For the mobile phase, we used water (solvent A) and methanol (solvent B) at a constant flow rate of 1 mL/min. The gradient started with 80–90% for solvent B at 15 min returning to 80% at 30 min. All sub-fractions were detected according to characteristic UV-vis spectra (spectral range of 200–450 nm) and retention times. Preparative HPLC was carried out using a preparative column Shimadzu PREP-ODS (H) (250 mm × 20 mm – i.d). To isolate the sub-fractions, a gradient using both solvents was used at room temperature with a flow rate of 8 mL/min [6]. Three sub-fractions were isolated from H-Fr.

### High resolution gas chromatography with flame ionization detection (HRGC-FID)

Fatty acid methyl esters (FAMEs) were prepared from EEP, its fractions and sub-fractions according to a modification of the method by Hartman and Lago (1973) [15]. Samples of 0.3 μL were injected into a Hewlett-Packard (HP) 5890 series II gas chromatographer equipped with a 60 m DB-23 (0.25 mm I.D., 0.25 μm film thickness) column. The oven temperature was programmed as follows: 130°C (1.0 min) to 170°C (6.5°C/min), 170°C to 215°C (2.75°C/min), 215°C (12 min), 215°C to 230°C (40°C/min), and 230°C (3 min). The injector and detector were used at 270°C and 280°C, respectively. FAMEs were identified using standard of fatty acids (Sigma, St Louis, MO, USA) with 6, 8, 10, 12, 14, 15, 16 (cis e trans), 17, 18 (cis e trans), 20, 22, and 24 atoms of carbon, saturated and unsaturated and helium (He) was used as the carrier gas (1.0 mL/min).

### Gas chromatography-mass spectrometry (GC-MS)

The analyses of fatty acid were performed after methylation of the EEP, its fractions, and sub-fractions as described in the literature [16]. Aliquots of 400 μL (10 mg/mL) of the samples were placed into glass vials and 400 μL of CH_2_N_2 _were added to each solution. All the samples were refrigerated for 4 h to allow complete methylation and then analyzed by GC-MS using a CBP5 column (30 m × 0.25 mm i.d.) installed in a GC 17A (Shimadzu Co.) instrument interfaced with a QP 5000 mass selective detector operated in scanning mode (m/z 40–400). For GC-MS analysis, the temperature was set from 50°C (0.3 min hold) to 285°C (15 min hold) at a rate of 6°C/min. The samples were injected with an AOC-17 autoinjector using a splitless injection technique (0.6 μL injection volume) and He flow was set at 1.0 mL/min. The GC-MS peaks were identified by comparison with data found in the literature and characterized using the library search software of Shimadzu Class-Vp (Wiley 138 and Nist 98 databases).

### Microbial susceptibility testing

The minimum inhibitory concentration (MIC) and minimum bactericidal concentration (MBC) of the EEP, fractions, pure fatty acids, and isolated sub-fractions were evaluated to determine their antimicrobial activity using *Streptococcus mutans *Ingbritt 1600 and *Staphylococcus aureus *ATCC 25923, based on previously published methodology [12,13]. To determine MIC we used inoculums of 5 × 10^5 ^CFU/mL, concentrations of EEP, fractions, and pure fatty acids ranging from 6.25 to 1600 μg/mL, and concentration of isolated sub-fractions ranging from 0.2 to 210 μg/mL. To determine MBC, aliquots of 20 μL of all incubated tubes with concentrations higher than the MIC were subcultured on BHI agar and supplemented with 5% defibrinated sheep blood using a spiral plater (Whittley Automatic Spiral Plater) [12]. All these analyses were performed in triplicate. We used ethanol (final ethanol concentration: 0.6%, v/v) as the control vehicle and digluconate chlorhexidine 0.12% (Sigma^®^) as positive control in both tests.

## Results and Discussion

Table 1 shows MIC and MBC values for EEP, H-Fr, and P-Fr, respectively. MIC values indicated that the H-Fr exhibited strong antibacterial activity against *S. aureus *and *S. mutans*, with concentrations as low as 25 μg/mL and 50 μg/mL, respectively. Similar results were found in other studies for *S. mutans *Ingbritt 1600 [12,13], but no data concerning antimicrobial activity of EEP and its fractions against *S. aureus *were available in the literature. The H-Fr showed lower values than the other samples and this finding indicated the presence of an active compound in this fraction. However, MIC and MBC values for EEP, H-Fr, and P-Fr were higher than that found for the positive control (chlorhexidine 0.12%). This might be explained by the fact that a synthetic pure mono-drug (chlorhexidine) was compared with the fractions of a natural product that presents its biological compound diluted in the samples (EEP, H-Fr, or P-Fr).

**Table 1:** Minimum inhibitory concentration (MIC) and minimum bacterial concentration (MBC) of different fractions against *Staphylococcus aureus *ATCC 25923 and *Streptococcus mutans *Ingbritt 1600.

Sample	*Staphylococcus aureus *ATCC 25923		*Streptococcus mutans *Ingbritt 1600	
	
	MIC (μg/mL)	MBC (μg/mL)	MIC (μg/mL)	MBC (μg/mL)
EEP	25–50	400–800	50–100	> 1600
H-Fr	25–50	200–400	50–100	800–1600
P-Fr	50–100	400–800	100–200	> 1600
Positive Control*	1.5–3.1	3.1–6.25	0.75–1.5	1.5–3.1

*Positive Control: digluconate chlorhexidine 0.12% – Sigma^®^.

ND: not determined.

Experiments carried out in triplicate.

**Figure 1:**
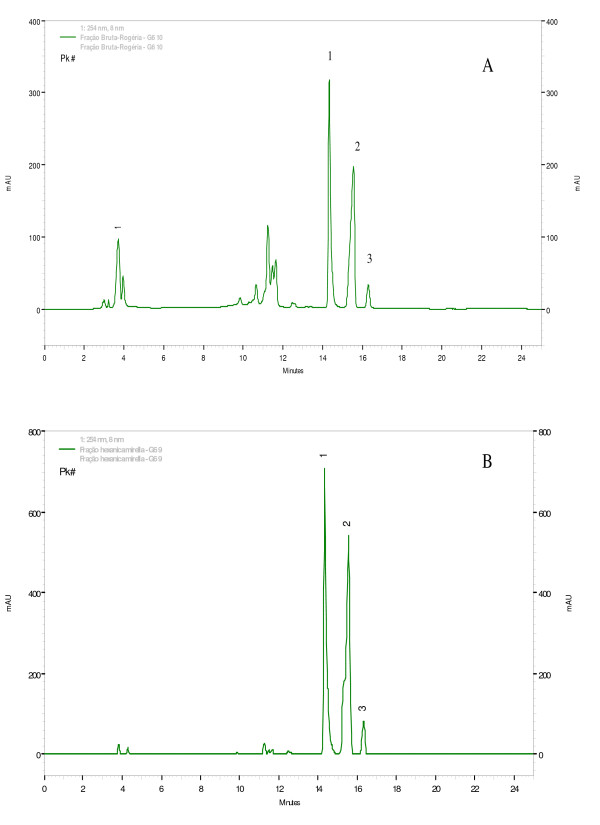
**RP-HPLC Chromatograms of propolis type 6**. (A) Ethanolic extract of propolis (EEP) and (B) hexane fractions (H-Fr). Peaks 1, 2, and 3 have the same retention time in both A and B graphics.

HPLC analyses of EEP and H-Fr demonstrated identical chemical profiles for both, since they presented three chemical peaks in common and in similar proportions (Figure 1) and neither flavonoids nor cinnamic acid derivatives were detected.

EEP and H-Fr chemical compositions were analyzed by HRGC-FID and GC-MS, and high and similar concentrations of fatty acids were observed, which is corroborated by previous findings (Table 2) [12,13]. We identified the following fatty acids: oleic (18:1), palmitic (16:0), linoleic (18:2), and stearic (18:0), present in higher concentrations in EEP and H-Fr, as already shown in previous studies [12,13]. Based on previous studies showing similar results [17-19], some authors suggested that the chemical composition of this propolis might be responsible for its antimicrobial activity [12,13].

**Table 2:** Chemical composition of fatty acids of EEP and H-Fr from Brazilian propolis type 6 obtained by HRGC-FID and GC-MS.

Retention time (min)	Carbon saturation*	Fatty acid	EEP (%)	H-Fr (%)
3.03	10:0	capric acid	4.61	traces
13.39	16:0	palmitic acid	29.05	35.44
17.37	18:0	estearic acid	3.12	4.41
17.59	18:1	oleic acid	55.72	50.33
18.54	18:2	linoleic acid	3.74	5.51
20.23	18:3	Alpha linolenic acid	1.47	1.98
21.28	22:0	behenic acid	2.29	2.33

*10:0 = decanoic acid; 16:0 = n-dodecanoic acid; 18:0 = n-octadecanoic acid; 18:1 = *cis*-9-octadecenoic acid; 18:2 = *cis*-9.12-octadecadienoic acid; 18:3 = *cis*-9.12.15-octadecatrienoic acid; 22:0 = docosanoic acid.

The same fatty acids (oleic, palmitic, linoleic, and stearic acid – Sigma^®^), tested isolatedly or mixed, showed no bacterial activity against Gram-positive bacteria (*S. aureus *or *S. mutans*) at concentrations lower than 1600 μg/mL. However, MIC values found for H-Fr were lower than 50 and 100 μg/mL for *S. aureus *and *S. mutans*, respectively (Table 1), suggesting that other compounds, different from the fatty acids tested, might account for the antimicrobial activity detected in the present study.

Since no satisfactory MIC and MBC results were found for the fatty acids tested, three unpurified sub-fractions were isolated from H-Fr by means of preparative HPLC and tested, isolatedly or mixed, for antibacterial activity. Only sub-fraction 1 (peak 1, Figure 1B) showed satisfactory antibacterial activity against *S. aureus *and *S. mutans*, with MIC ranging from 1.56 to 3.12 and 3.12 to 6.2 μg/mL, respectively (Table 3), but the mixture of these three sub-fractions showed no bacterial activity. This might have happened due to the dilution of the active compounds present in sub-fraction 1, thus excluding the possibility of any synergistic effects of these three sub-fractions.

**Table 3:** Minimum inhibitory concentration (MIC) and minimum bacterial concentration (MBC) of three isolated sub-fractions against *Staphylococcus aureus *ATCC 25923 and *Streptococcus mutans *Ingbritt 1600.

Sample	*Staphylococcus aureus *ATCC 25923		*Streptococcus mutans *Ingbritt 1600	
	
	MIC (μg/mL)	MBC (μg/mL)	MIC (μg/mL)	MBC (μg/mL)
Sub-fraction 1	1.5–3.1	26–53	3.1–6.25	53–106
Sub-fraction 2	> 210	> 210	> 210	> 210
Sub-fraction 3	> 210	> 210	> 210	> 210
Positive control*	1.5–3.1	3.1–6.25	0.75–1.5	1.5–3.1

*Positive control: digluconate chlorhexidine 0.12% – Sigma^®^.

Experiments carried out in triplicate.

**Figure 2:**
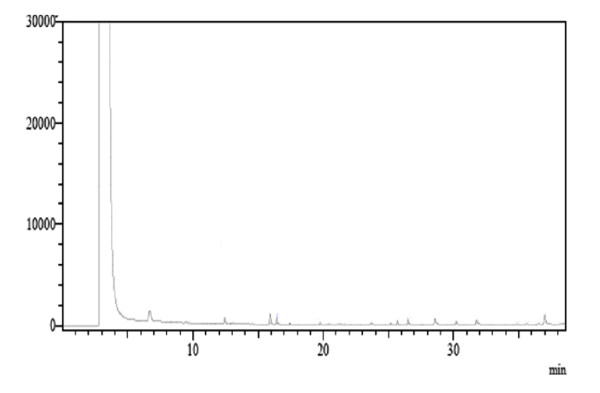
**Profile of the isolated sub-fraction 1 in HRCG-FID**.

**Figure 3:**
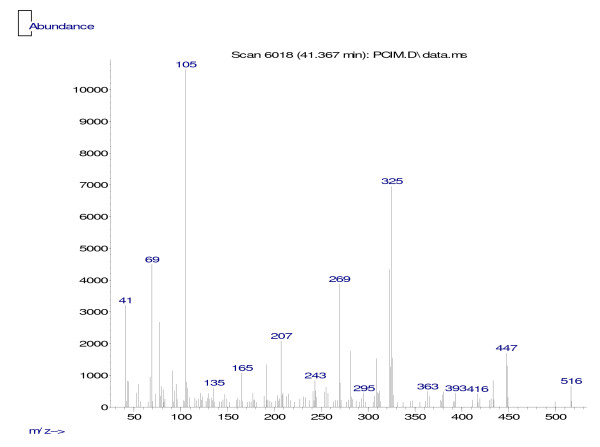
**Mass spectra peaks (m/z) of sub-fraction 1 obtained by GC-MS**.

MIC values found for sub-fraction 1 were similar to those obtained for the positive control (chlorhexidine 0.12%) for both microorganisms tested, suggesting that sub-fraction 1 might contain an antimicrobial bioactive compound. In addition, since chlorhexidine, a pure mono-drug, and sub-fraction 1, an impure bioactive compound, showed similar MIC results, we might speculate that this compound could be a promising antimicrobial agent, which needs to be isolated and identified. TLC and HPLC analyses of sub-fraction 1 showed a mixture of non-polar compounds; however, when this sub-fraction was analyzed by HRGC-FID, no fatty acid characteristics were found (Figure 2). Furthermore, when sub-fraction 1 was analyzed by PDA detector, two maximum absorbance values were found (240 nm and 300 nm), which is characteristic of benzophenone compounds [20,21]. GC-MS analyses showed fragments at m/z 105, indicating that this sub-fraction belongs to the benzophenone class and is not a fatty acid, as suggested in the literature [10,12,13]. Fragments at m/z 77, 69, and 55 showed its prenylated nature (Figure 3) and those at m/z 433 and 309 confirmed that this is a polyprenylated benzophenone [21].

Benzophenones are among the most important chemical compounds identified and isolated from propolis found in tropical regions [20-23]. They have been reported as having various biological activities, such as potential antimicrobial activity against both Gram-positive and Gram-negative bacteria [22-27].

## Conclusion

No antimicrobial activity was observed for the main fatty acids identified in Brazilian propolis type 6. Sub-fraction 1 isolated from this type of propolis belongs to the benzophenone class and was responsible for the antimicrobial activity observed in the present study. The identification of this bioactive compound will improve the development of more efficient uses of this natural product.

## Competing interests

The authors declare that they have no competing interests.

## Authors' contributions

This study was conceived, directed, and coordinated by PLR and SMA, who also critically revised the manuscript with the help of MI. MLC participated throughout the study (chemical and microbiological assays). WR and RCZ worked in the preparation of the extracts, fractions, and analyses of fatty acids. VLR and MAF gave their support in the chromatography laboratory and helped interpret data. All the authors read and approved the final manuscript.

## Pre-publication history

The pre-publication history for this paper can be accessed here:



## References

[B1] Ghisalberti EL (1979). Propolis: a review. Bee World.

[B2] Burdock GA (1998). Review of the biological properties and toxicity of bee propolis. Food Chem Toxicol.

[B3] Park YK, Alencar SM, Aguiar CL (2002). Botanical origin and chemical composition of Brazilian propolis. J Agric Food Chem.

[B4] Bankova V (2005). Recent trends and important developments in propolis research. Evid Based Complement Alternat Med.

[B5] Castro ML, Duarte S, Ikegaki M, Cury JA, Koo H, Alencar SM, Rosalen PL (2007). Própolis do sudeste e nordeste do Brasil: influência da sazonalidade na atividade antibacteriana e composição fenólica. Quím Nova.

[B6] Alencar SM, Oldoni TLC, Castro ML, Cabral ISR, Costa-Neto CM, Cury JA, Rosalen PL, Ikegaki M (2007). Chemical composition and biological activity of a new type of Brazilian propolis: red propolis. J Ethnopharmacol.

[B7] Koo H, Gomes BP, Rosalen PL, Ambrosano GM, Park YK, Cury JA (2000). In vitro antimicrobial activity of propolis and *Arnica montana *against oral pathogens. Arch Oral Biol.

[B8] Bankova VS, Castro SL, Marcucci MC (2000). Propolis: recent advances in chemistry and plant origin. Apidologie.

[B9] Marcucci MC, Ferreres F, García-Viguera C, Bankova VS, Castro SL, Dantas AP, Valente PHM, Paulino N (2001). Phenolic compounds from Brazilian propolis with pharmacological activities. J Ethnopharmacol.

[B10] Park YK, Ikegaki M, Alencar SM, Moura FF (2000). Evaluation of Brazilian propolis by both physicochemical methods and biological activity. Honeybee Sci.

[B11] Koo H, Rosalen PL, Cury JA, Ambrosano GMB, Murata RM, Yatsuda R, Ikegaki M, Alencar SM, Park YK (2000). Effect of a new variety of Apis mellifera propolis on mutans streptococci. Curr Microbiol.

[B12] Duarte S, Koo H, Bowen WH, Hayacibara MF, Cury JA, Ikegaki M, Rosalen PL (2003). Effect of a novel type of propolis and its chemical fractions on glucosyltransferases and on growth and adherence of mutans streptococci. Biol Pharm Bull.

[B13] Duarte S, Rosalen PL, Hayacibara MF, Cury JA, Bowen WH, Marquis RE, Rehder VL, Sartoratto A, Ikegaki M, Koo H (2006). The influence of a novel propolis on mutans streptococci biofilmes and caries development in rats. Arch Oral Biol.

[B14] Tanaka JCA, Silva CC, Dias Filho BP, Nakamura CV, Carvalho JE, Foglio MA (2005). Constituintes químicos de *Luehea divaricata *Mart (Tiliaceae). Quím Nova.

[B15] Hartman L, Lago RC (1973). Rapid preparation of fatty acid methyl esters from lipids. Lab Pract.

[B16] Markham KR, Mitchell KA, Wilkins AL, Daldy JA, Lu Y (1996). HPLC and GC-MS identification of the major organic constituints in New Zealand propolis. Phytochemistry.

[B17] Dilika F, Bremner PD, Meyer JJM (2000). Antibacterial activity of linoleic and oleic acids isolated from Helichrysum pedunculatum: a plant used during circumcision rites. Fitoterapia.

[B18] Wille JJ, Kydonieus A (2003). Palmitoleic acid isomer (C16: 1 Delta 6) in human skin sebum is effective against gram-positive bacteria. Skin Pharmacol Appl Skin Physiol.

[B19] Yang CM, Luedecke LO, Swanson BG, Davidson PM (2003). Inhibition of microorganisms in salad dressing by sucrose and methylglucose fatty acid monoesters. J Food Process Preservat.

[B20] Hernández IM, Fernandez MC, Cuesta-Rubio O, Piccinelli AL, Rastrelli LJ (2005). Polyprenylated benzophenone derivatives from Cuban propolis. J Nat Prod.

[B21] Tomas-Barberan FA, Garcia-Viguera C, Vit-Olivier P, Ferreres F, Tomas-Lorente F (1993). Phytochemical evidence for the botanical origin of tropical propolis from Venezuela. Phytochemistry.

[B22] Cuesta Rubio O, Cuellar Cuellar A, Rojas N, Velez Castro H, Rastrelli L, Aquino R (1999). A polyisoprenylated benzophenone from Cuban propolis. J Nat Prod.

[B23] Cuesta-Rubio O, Frontana-Uribe BA, Ramírez-Apan T, Cárdenas J (2002). Polyisoprenylated benzophenones in Cuban propolis; biological activity of nemorosone. Z Naturforsch [C].

[B24] Trusheva B, Popova M, Bankova V, Simova S, Marcucci MC, Miorin PL, Pasin FR, Tsvetkova I (2006). Bioactive constituents of Brazilian red propolis. Evid Based Complement Alternat Med.

[B25] Bakana P, Claeys M, Totté J, Pieters LA, Van Hoof L, Tamba-Vemba L, Berghe DA Van den, Vlietinck AJ (1987). Structure and chemotherapeutical activity of a polyisoprenylated benzophenone from the stem bark of Garcinia huillensis. J Ethnopharmacol.

[B26] Hussain RA, Owegby AG, Parimoo P, Waterman PG (1982). Kolanone, a novel polyisoprenylated benzophenone with antimicrobial properties from the fruit of *Garcinia kola*. Planta Med.

[B27] Iinuma M, Tosa H, Tanaka T, Kanamaru S, Asai F, Kobayashi Y, Miyauchi K, Shimano R (1996). Antibacterial activity of some Garcinia benzophenone derivatives against methicillin-resistant *Staphylococcus aureus*. Biol Pharm Bull.

